# High-density polyethylene crystals with double melting peaks induced by ultra-high-molecular-weight polyethylene fibre

**DOI:** 10.1098/rsos.180394

**Published:** 2018-07-18

**Authors:** Weijun Miao, Hao Zhu, Tianchen Duan, Hongbing Chen, Feng Wu, Libin Jiang, Zongbao Wang

**Affiliations:** Ningbo Key Laboratory of Specialty Polymers, Faculty of Materials Science and Chemical Engineering, Ningbo University, Ningbo 315211, People's Republic of China

**Keywords:** high-density polyethylene, ultra-high-molecular-weight polyethylene fibre, epitaxial crystallization, double melting peaks

## Abstract

High-density polyethylene (HDPE)/ultra-high-molecular-weight polyethylene (UHMWPE) fibre composites were prepared via solution crystallization to investigate the components of epitaxial crystal growth on a highly oriented substrate. Scanning electron microscopy morphologies of HDPE crystals on UHMWPE fibres revealed that the edge-on ribbon pattern crystals that were formed initially on UHMWPE fibres converted afterwards to a sheet shape as crystallization progressed. Wide-angle X-ray diffraction confirmed that the polymer chain oriented along the fibre axis and the orthorhombic crystal form of HDPE remained unchanged in HDPE/UHMWPE fibre composite systems. The thermal behaviour of the fibre composites measured by differential scanning calorimetry showed double melting peaks, the nature of which, as disclosed by partial melting experiments, is ascribed to bilayer components existing in the induced crystals: the inner layer is composed of more regularly folded chain crystals induced by UHMWPE fibres, and the outer layer formed on the inner one with a thinner and lower ordered crystal structure.

## 1. Introduction

Polymeric materials have the great advantage that they meet the requirements for many desired applications; this stems from the fact that the primary and aggregation structures of polymers can be effectively manipulated as needed. Significantly, the physical properties of polymeric materials are susceptible to being influenced by external factors. For example, in a polymer composite system, the improvement in the mechanical properties of the polymers can be attributed not only to the mechanical properties of the reinforcements, but also to the polymer crystallization, which can be influenced by reinforcements [1–5]. Reinforcements as a heterogeneous nucleation agent decrease the surface free energy barrier for polymer nucleation, and then change the crystallinity, spherulite size and crystal orientation, which has a profound effect on the ultimate properties of the polymer matrix. Therefore, this study of the interfacial crystallization of polymers is an important part of regulating the overall performance of polymer composites.

Fibre-reinforced polymer composites offer a number of potential advantages, such as stiffness, tensile strength and heat distortion temperature [6,7], compared with traditional materials. It has been proposed that this improvement may result from changes in the morphology and crystallinity of the polymer matrix in the interfacial region. In particular, when heterogeneous nucleation occurs with sufficiently high density along a fibre surface, the resulting crystal growth is restricted to the lateral direction, so that a columnar layer develops around the fibre, a phenomenon known as transcrystallization. This nucleation of a transcrystallized region around the reinforcing fibre is thought to be central to the improvement of some composite properties [8,9]. The formation mechanism of transcrystallization strongly depends on the chemical composition of the fibre surface [10]. For the crystalline polymer fibres, highly oriented molecular chains induce polymer epitaxy and lattice matching between the polymer, and the oriented fibre crystals provide a favourable situation for transcrystallization. For example, the excellent matching of nylons and isotactic polypropylene (iPP) with high-modulus graphitic carbon fibres has led to the transcrystallization of nylons and iPP around the carbon fibres [11]. Many studies on the mechanism of transcrystallization have been carried out over the past few decades [12–16], but the melting behaviour of the crystallization structure induced by the oriented polymer substrate has been investigated only rarely [17–26]. Petermann and colleagues [24–26] studied the components of crystals (kebab) grown epitaxially on the surface of an oriented molecular chain (shish) formed in a shear field. Their results confirmed the existence of several components in the kebab structure, including the partially extended-chain microkebabs and the overgrown macrokebabs. The macrokebabs grown on the microkebab templates were less thermally stable than the microkebabs and, upon heating, became segmented and discontinuous. It was thought that microkebabs originated from the cilia that were firmly attached to the shish backbone, parts of which were intrinsically implanted in the shish. Therefore, components of the kebab formed in shearing flow cannot represent those crystals producted by epitaxy because of the chain entanglement in the shear field. None of the current research involves the component gradient induced by the oriented substrate. It is of great significance to analyse the crystal constituent, which is beneficial to obtaining a full understanding of orientation-induced crystallization.

In this paper, we pay close attention to the components of crystals grown epitaxially on the surface of an oriented substrate. A polyethylene/ultra-high-molecular-weight polyethylene (UHMWPE) fibre composite is employed as an example. The UHMWPE fibre is also known as an extended-chain polyethylene fibre, as the high degree of chain orientation due to the gel-spinning process gives the fibre a high ability to induce polymer crystallization. We use high-density polyethylene (HDPE), which is a polymeric model material due to its simple chemical structure and well-known properties [27,28]. The pure carbon backbone of polyethylene (PE) rules out the interference of heteroatoms and intramolecular and intermolecular forces on crystallization, which is beneficial to the study of interfacial crystallization. Also, both the fibre and the matrix are made up of the same polymer (single-polymer matrix/fibre composites), also known as self-reinforced single-polymer composites, which is conducive to surface-induced crystallization because of the identical chemical composition and perfect lattice matching. Therefore, a HDPE/UHMWPE fibre composite is a useful model to investigate the crystal components produced by epitaxial crystallization. On the other hand, solution crystallization was employed in this study to produce HDPE crystals induced by UHMWPE fibres, which can completely remove the impacts of ontology crystallization and enable the morphology and structure of crystals formed on external interfaces to be studied [29–32]. The morphology of crystals formed on UHMWPE fibres was clearly revealed by scanning electron microscopy (SEM), and the orientation of the polymer chain induced by UHMWPE fibres was determined by wide-angle X-ray diffraction (WAXD). The melting temperature (*T*_m_) values shown by differential scanning calorimetry (DSC) reflected the components of the crystals grown epitaxially on the surface of the UHMWPE fibres. This study will establish a better understanding of the influence of an oriented substrate on polymer crystallization, so as to provide guidance to modulate the physical properties of polymer/ fibre composites.

## 2. Experimental section

### 2.1. Materials

The HDPE was purchased from the Dow Chemical Company, with an average weight *M*_n_ = 15 820 g mol^−1^ and a polydispersity index *M*_w_*/M*_n_ = 4.9. The UHMWPE resin used in this study has a viscosity-average molecular weight (*M*_v_) of 2.7 × 10^6^, which was supplied by the Sinopec Beijing Yanshan Company.

### 2.2. Sample preparation

The UHMWPE gels with a concentration of 8 wt% were prepared by suspending UHMWPE resin in paraffin oil through a twin-screw extruder at approximately 150–250°C. The UHMWPE gels were then used for gel spinning with a 240 conical die spinneret plate. The exit diameter, extrusion rate and extrusion temperature were 1 mm, 8 m min^−1^ and 250°C, respectively. The cold stretching process with a stretch ratio of 4 was performed at room temperature after fibres were spun from the spinneret plate. Then, gel-spinning fibres were put through a dichloromethane bath to extract paraffin oil. Finally, the fibres were put through three hot-drawing stages at temperatures of 110°C, 120°C and 130°C, respectively.

**Scheme 1.**
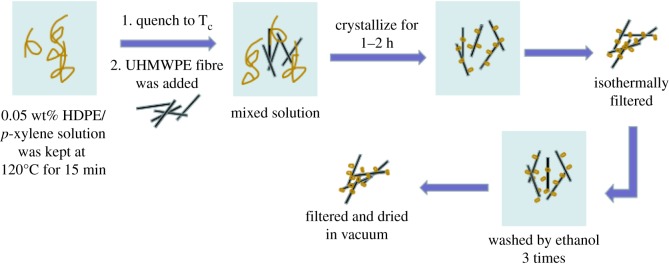
Fabrication process of HDPE/UHMWPE fibre composites.

The fabrication process of HDPE/UHMWPE fibre composites is illustrated in scheme 1. HDPE/*p*-xylene solution was prepared by dissolving HDPE into *p*-xylene at 120°C with stirring at 1200 r.p.m. for 15 min, where the mass concentration of HDPE was controlled at 0.05 wt%. Fibres with the same mass as HDPE were cut into 3 cm long staple fibres by the blade, and subsequently added into the HDPE/*p*-xylene solution. Then, the solution was quenched to the pre-set crystallization temperature *T*_c_ within 1 min. After that, the stirring rate was decreased to 400 r.p.m. to avoid destroying the HDPE crystals deposited on the fibres. The crystallization time was controlled to be approximately 1–3 h and the stirring was stopped. The sample was isothermally filtered after crystallization to remove the uncrystallized materials. Subsequently, fibre composites were transferred into ethanol (25°C). After being washed with ethanol carefully three times, the composites were dried at 50°C under a vacuum for approximately 36–48 h.

### 2.3. Characterization

SEM (S4800) was used to observe the surface morphologies of the UHMWPE fibre composites. All the specimens were coated with gold to avoid charging. Inevitably, the size values calculated based on the scanning electron micrographs are on the high side; this is caused by the gold particles aggregating on the surface of the samples.

WAXD measurements were performed with a Xeuss WAXS system (Xenocs) at Ningbo University, China. The wavelength of the X-ray radiation was 0.154 nm. Two-dimensional (2D) WAXD patterns were collected with a MAR345 X-ray detector. Two scatter-less slits (Xenocs) were used to depress the parasitic scattering. The sample holder was mounted onto an optical table. The sample-to-detector distance was 262 mm for WAXD. The WAXD image acquisition time of each data frame was 1800 s. All X-ray images were corrected for background scattering, air scattering and beam fluctuations. The WAXD measurement data analysis was carried out by the Fit2d software package [33]. The degree of crystal orientation in the fibre composites was calculated by Herman's method [34]. Accordingly, the crystalline orientation can be characterized by the average orientation of the normal to the crystalline plane with respect to an external reference frame. The fibre axis direction was taken as the reference direction. For a set of (*hkl*) planes, the average orientation, expressed as ${\left(\cos^{2}\phi \right)}_{hkl}$, can be calculated mathematically using

2.1 $${\left(\cos^{2}\phi \right)}_{hkl}=\frac{\int_{0}^{\pi /2}I(\phi )\sin\phi \cos^{2}\phi \;\text{d}\phi }{\int_{0}^{\pi /2}I(\phi )\sin\phi \;\text{d}\phi },$$

where ϕ is the azimuthal angle and I(ϕ) is the scattered intensity along the angle ϕ. Herman's orientation function *f* is defined as

2.2 $$f=\frac{3{\left(\cos^{2}\phi \right)}_{hkl}-1}{2}.$$

The value of Herman's orientation function *f* is 0 when the lamellar direction distribution is isotropic, and the value is 1 when the lamellae are parallel to the reference direction ($\phi =0^{o}$). The degree of orientation, *f*_110_, is calculated from the azimuthal intensity distribution, I(ϕ), of the (110) reflection of crystals in various composite samples.

DSC experiments were carried out using a Perkin-Elmer DSC8000. The samples with an average weight of approximately 2–4 mg were heated from 30°C to 180°C at a scanning rate of 10°C min^−1^ under a nitrogen atmosphere and were cooled and reheated at the same rate. For fibre composite samples, the first heating curves were collected for analysis.

## 3. Results and discussion

### 3.1. Surface morphologies of HDPE crystallized on UHMWPE fibres

**Figure 1.**
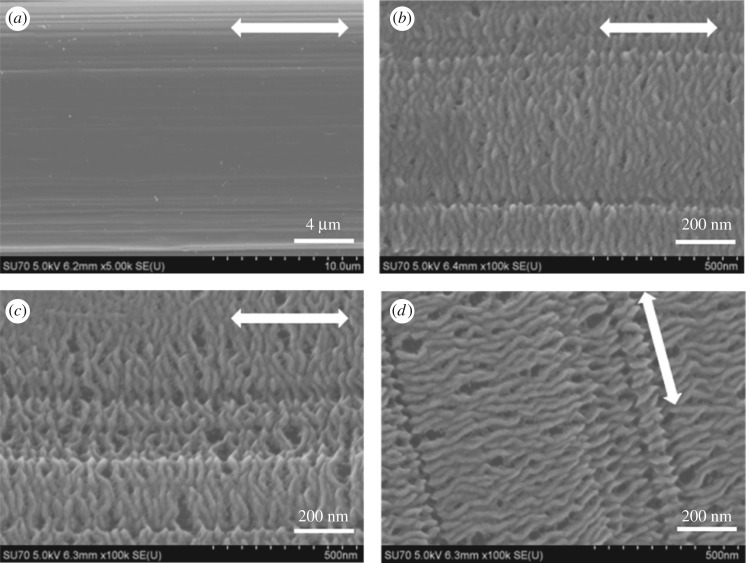
SEM micrographs of UHMWPE fibres in *p*-xylene at 110°C for 1 h (*a*) and the crystallization of HDPE on UHMWPE fibres in *p*-xylene at (*b*) 90°C, (*c*) 100°C and (*d*) 110°C for 1 h. The arrow represents the orientation of the UHMWPE fibres.

The surface morphologies for the UHMWPE fibres before and after crystallizing HDPE were verified by SEM as shown in figure 1. The virgin UHMWPE fibres with a neat surface are shown in the electronic supplementary material, figure S1, the surface structure of which remains unchanged after being soaked in *p*-xylene at 110°C for 1 h (figure 1*a*). Therefore, we can confirm that the solvent has no influence on the surface structure of UHMWPE fibres. The HDPE/UHMWPE fibre crystals were obtained by HDPE crystallization on UHMWPE fibres in *p*-xylene at a concentration of 0.05 wt% at different crystallization temperatures. Figure 1*b,c* shows that the HDPE crystals periodically decorate the fibres with long-range order structures and form a ribbon pattern at all experimental temperatures. The average sizes of the ribbon crystals formed on the fibres are listed in table 1 on the basis of measurement of 300 lamellae. The lateral dimensions (*d*) of the HDPE crystal growth on the UHMWPE fibres were calculated using

3.1 $$d=\frac{D_{\mathrm{composite}}-D_{\mathrm{fibre}}}{2}.$$

Here, $D_{\mathrm{composite}}$ is the diameter of the HDPE/UHMWPE fibre composite and $D_{\mathrm{fibre}}$ is the diameter of the UHMWPE fibre, calculated to be 17.2 µm, as shown in the electronic supplementary material, figure S1.

**Table 1.** Average size of HDPE crystals formed on UHMWPE fibres based on the SEM images of 300 lamellae.

crystallization temperature (°C)	thickness of crystal (nm) after 1 h	*d*_1_ (nm)^a^	thickness of sheet (nm) after 3 h	*d*_2_ (nm)^b^
90	14.1 ± 0.7	330 ± 9	14.7 ± 1.1	610 ± 15
100	14.9 ± 0.7	370 ± 11	15.1 ± 0.8	690 ± 17
110	15.3 ± 0.8	190 ± 8	15.7 ± 1.1	430 ± 12

^a^*d*_1_ is the lateral dimension of HDPE crystallization on UHMWPE fibres for 1 h.

^b^*d*_2_ is the lateral dimension of HDPE crystallization on UHMWPE fibres for 3 h.

The lateral dimension of the ribbon crystal (*d*_1_) is about 330 ± 9, 370 ± 11 and 190 ± 8 nm, for 90°C, 100°C and 110°C, respectively (table 1). It first increases and then decreases with the increase in crystallization temperature. The main reasons for this are as follows: for the sample crystallized at the lower temperature (90°C), heterogeneous nucleation induced by fibres and self-nucleation of HDPE are almost simultaneous due to the higher rate of undercooling and the high density of the nucleus causes more crystal lamellae to form on the surface of the fibres, which results in the ribbon crystals packing closely along the fibre axis as shown in figure 1*b*. Moreover, competition between the heterogeneous nucleation and self-nucleation suppresses the growth of the ribbon crystals on the fibres, which results in a smaller lateral dimension of the HDPE crystal lamellae. With an increase in crystallization temperature, heterogeneous nucleation plays a major role and the diameter of the ribbon crystals increases to a maximum of 370 ± 11 nm at 100°C. However, as the crystallization temperature increases successively, the average diameter of the ribbon crystals decreases to 190 ± 8 nm at 110°C, which can be attributed to the lower growth rate of the crystals at higher crystallization temperatures. On the contrary, the interval of the ribbon crystals continues to increase as the crystallization temperature increases (figure 1*c,d*). This is because the unstable crystal nucleus gradually disappears as the degree of undercooling decreases. The average thickness of the ribbon crystals increases as the crystallization temperature increases, from 14.1 ± 0.7 nm at 90°C to 15.3 ± 0.8 nm at 110°C (table 1). It is known that crystal thickness is strongly dependent on crystallization temperature. Consequently, lamellae formed at different temperatures have different thicknesses. From the above-mentioned data, it can be concluded that the crystallization temperature has a significant influence on the interval and thickness of the ribbon crystals formed on UHMWPE fibres.

**Figure 2.**
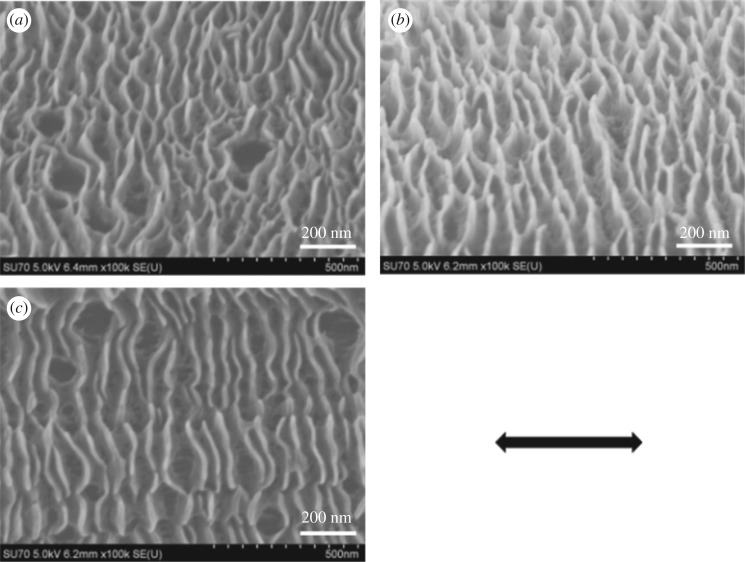
SEM micrographs of the HDPE crystals on UHMWPE fibres in *p*-xylene at (*a*) 90°C, (*b*) 100°C and (*c*) 110°C for 3 h. The arrow represents the orientation of the UHMWPE fibre.

The evolution of the ribbon crystals with crystallization time was also studied. The morphologies of HDPE crystals on UHMWPE fibres in *p*-xylene for 3 h are shown in figure 2. The average sizes of the crystals are listed in table 1. We can clearly see from figure 2 that the ribbon pattern is converted to an edge-on sheet shape after 3 h crystallization. If crystallization is continued for a further 2 h, the lateral dimension of the crystals (*d*_2_) increased by almost two times compared with that for isothermal crystallization for 1 h. This suggests that the lateral growth speed of the HDPE chains along the fibres is rapid. According to *d*_2_, as shown in table 1, the lateral growth rate of the crystals also decreased after an initial increase with increasing crystallization temperature: the growth rate increased initially from 140 nm h^−1^ at 90°C to 160 nm h^−1^ at 100°C, then decreased to 110 nm h^−1^ at 110°C. This further indicates that both high temperatures and low temperatures are not beneficial for the lateral growth of crystals on UHMWPE fibres. Moreover, the lateral growth of crystals as the crystallization time increases produces more curved lamellae (figure 2) than crystallization for 1 h (figure 1), suggesting that the regularity of the crystal becomes lower after 3 h crystallization. This may be because the induction of the fibres is greatly weakened away from the highly oriented surface with the growth of HDPE crystals. It can also be observed from table 1 that the thickness of the sheet remains stable at identical crystallization temperatures. For instance, the average thickness of crystals is 14.1 ± 0.7 nm and 14.7 ± 1.1 nm, respectively, after 1 h and 3 h at 90°C as shown in table 1. This also indicates that the thickness of the crystals is directly related to the crystallization temperatures at a later growth stage. Additionally, the HDPE molecular chains are extensively consumed after a long period of crystallization and the rest of the molecular chains can only meet the need for partial growth of the lamellae. Therefore, we can also find that the interval of the sheet crystals becomes larger after crystallization for another 2 h by comparing figures 1 and 2.

### 3.2. Orientation of HDPE/UHMWPE fibre composites

**Figure 3.**
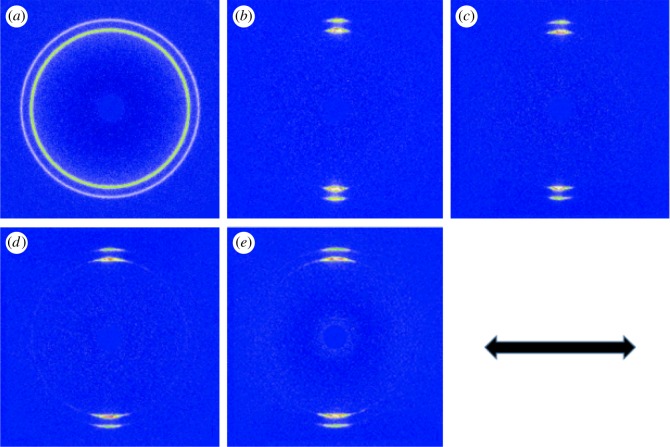
2D-WAXD patterns of neat HDPE (*a*), UHMWPE fibres (*b*) and their composites produced at (*c*) 110°C, (*d*) 100°C and (*e*) 90°C in *p*-xylene for 1 h. The arrow represents the orientation of the UHMWPE fibre.

**Figure 4.**
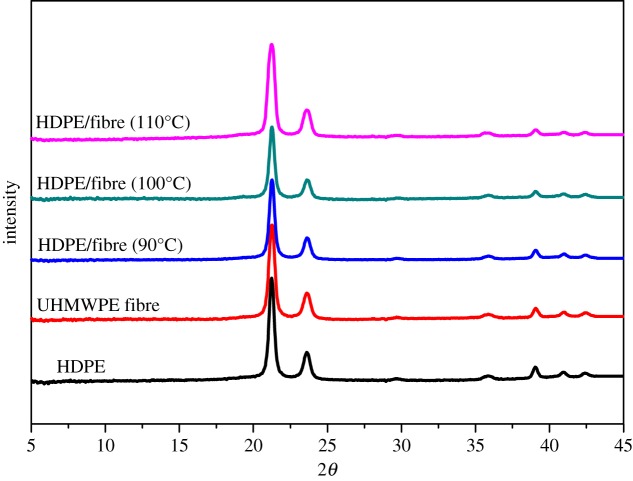
WAXD curves of neat HDPE, UHMWPE fibre and HDPE/UHMWPE fibre composites.

2D-WAXD was used to investigate the crystalline orientation of HDPE/UHMWPE fibre composites. Figure 3 shows the 2D-WAXD patterns of neat HDPE, UHMWPE fibres and HDPE/UHMWPE fibre composites. For neat HDPE, two isotropic circles can be seen in figure 3*a*; from the inner circle outwards, the diffraction circles are designated to the (110) plane and (200) plane of HDPE orthorhombic crystals. While the diffraction patterns correspond to the (110) and (200) orthorhombic crystalline planes of the UHMWPE fibres, two strong diffraction focused points appear, as shown in figure 3*b*, which also reflects the extremely high degree of chain orientation composed mostly of extended-chain crystals along the fibre axis. The effect of the fibres on the structural orientation can be estimated by comparing the 2D-WAXD patterns. The point diffraction patterns of the UHMWPE fibres change very little in fibre composites produced at 110°C for 1 h (figure 3*c*), which suggests that the chain orientation in HDPE crystals formed on UHMWPE fibres is parallel highly to the fibre axis. Therefore, a high degree of chain orientation of UHMWPE fibres is also maintained despite the HDPE crystals' epitaxial growth on them. The diffraction patterns corresponding to the (110) and (200) planes of the fibre composites change from a point to an arc when at 100°C for 1 h (figure 3*d*), and the size of the arcs becomes larger with decreasing crystallization temperature (figure 3*e*). The above variations in the patterns demonstrate an enhancement of the crystalline orientation of HDPE induced by UHMWPE fibres with increasing crystallization temperature. The corresponding WAXD curves of samples collected from 2D-WAXD patterns are shown in figure 4, which presents the same two typical diffraction peaks at 2*θ* = 21.2° and 23.6° corresponding to the (110) and (200) orthorhombic crystalline planes, respectively, suggesting that the crystal structure of HDPE remains unchanged despite being induced by UHMWPE fibres with high chain orientation.

**Figure 5.**
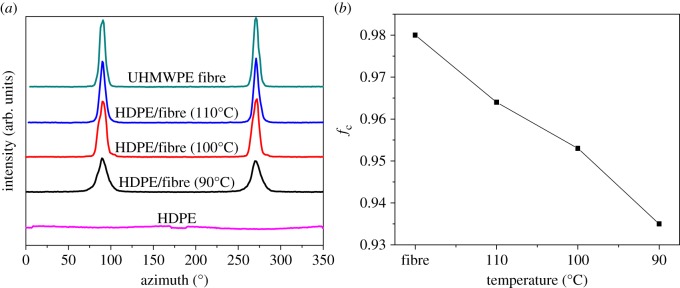
The diffraction intensity of the (110) plane as a function of the azimuthal angle (*a*) and the *f*_c_ as a function of the crystallization temperature (*b*).

Figure 5*a* shows the diffraction intensity of the (110) plane as a function of the azimuthal angle. No obvious peaks are observed in neat HDPE, which implies a random orientation of bulk HDPE crystals. For HDPE/UHMWPE fibre composites, the reflection intensity of the (110) plane along the fibre axis direction is enhanced and two peaks appear at 90° and 270°, indicating that the HDPE crystals are obviously oriented on the surface of the fibres compared with neat ones. Moreover, a higher half-peak width of reflection intensity for the sample produced at 90°C compared with that at higher temperature suggests a lower degree of crystal orientation. Figure 5*b* shows Herman's orientation parameter (*f*_c_) of neat fibre and its composites, which quantitatively express the improvement in the degree of orientation. UHMWPE fibres display an exceptionally high degree of crystal orientation: *f*_c_ is as high as 98%. After the epitaxial growth of HDPE crystals, the *f*_c_ of fibre composites produced at 110°C reduces slightly to 96.4%. Subsequently, *f*_c_ decreases with decreasing crystallization temperature, from 95.3% at 100°C to 93.5% at 90°C. These results indicate that the low crystallization temperature impaired the oriented arrangement and crystallization of HDPE molecular chains on UHMWPE fibres, which corresponds to the SEM results in figure 1.

For the samples crystallizing for 3 h, the diffraction arcs corresponding to the (110) and (200) planes of the fibre composites become larger as the crystallization time goes on. Taking the 2D-WAXD pattern of HDPE/UHMWPE fibre composite produced at 100°C for 3 h as an example (electronic supplementary material, figure S2), the size of the arcs is evidently larger than that for 1 h (figure 3*d*). And Herman's orientation parameter (*f*_c_) is 84.5%, which is significantly lower than that for 1 h (95.3% in figure 5*b*). This suggests that the orientation of the crystal becomes worse with increasing crystallization time, which is consistent with the curved lamellae shown in figure 2.

### 3.3. Melting behaviours of HDPE/UHMWPE fibre composites

**Figure 6.**
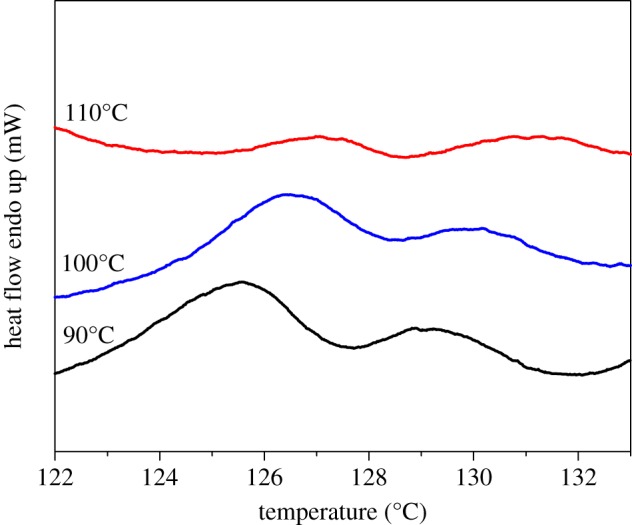
DSC first heating curves of HDPE/UHMWPE fibre composites produced at different temperatures in *p*-xylene for 1 h.

The melting behaviours give further insight into the unique crystallization mode of HDPE/UHMWPE fibre composites, and the first heating curves measured by DSC are shown in figure 6. The thickness of the lamellar crystal (*L*_c_) can be calculated from the melting temperature (*T*_m_) based on the Gibbs–Thomson equation [35],

3.2 $$L_{c}=\frac{2\sigma_{e}T_{m}}{\Delta H_{m}^{0}(T_{m}^{0}-T_{m})},$$

where *L*_c_ is the lamellar thickness, *σ*_e_ is the surface free energy of the folding surface, $T_{m}^{0}$ is the equilibrium melting temperature and $\Delta H_{m}^{0}$ is the fusion enthalpy of a perfect PE crystal with 100% crystallinity. The value of *σ*_e_ is 0.09 J m^−2^ for PE, and $T_{m}^{0}$ and $\Delta H_{m}^{0}$ are 414.6 K and 293 J cm^−3^, respectively [36,37].

For the neat UHMWPE fibre, the melting point (greater than 145°C) is much higher than that of bulk HDPE (131°C) because of the high degree of chain orientation (electronic supplementary material, figure S3). Surprisingly, the heating curves shown in figure 6 reveal unusual melting behaviours for these composites. Double melting peaks emerge in all HDPE crystals induced by UHMWPE fibres. For example, the composite produced at a crystallization temperature of 90°C has two melting points, 125.6°C and 129.4°C. Moreover, melting points deviate to high temperature with increasing crystallization temperature. For instance, melting points of the composite produced at a crystallization temperature of 100°C locate at 126.4°C and 130.1°C, which shift to higher values, 127.1°C and 131.2°C, for the sample produced at 110°C. Lamellar thicknesses (*L*_c_) calculated using equation (3.2) are listed in table 2, and are significantly lower than those obtained by SEM measurement, as shown in figure 1 and table 1. A higher SEM result may come from the fact that the gold coating on the surface of SEM specimens enhances the thickness and reduces the measurement precision. To explain the double melting peak phenomenon, we propose three possible mechanisms. (i) The melting of the smaller imperfect crystals, followed by recrystallization to form more perfect and larger crystals, and eventually the melting of these larger crystals is at a higher temperature. (ii) There exist two different structures in the HDPE crystals formed on the surface of UHMWPE fibres. Namely, one crystal form melted at low temperatures, and the other form corresponding to higher order crystal lattices melted at high temperatures. (iii) There exist two kinds of crystals with different thicknesses. Under the same heating conditions, crystals of identical polymer generated after recrystallization should have the same melting points. That is, higher melting temperature peaks of HDPE crystals formed on the surface of fibres at different crystallization temperatures should locate at same positions after melting recrystallization. This is clearly not consistent with figure 6. This primarily proves that double melting peaks are not caused by the melting–recrystallization mechanism. To further confirm the above hypothesis, melting behaviours at different heating rates were measured. Take the composite produced at a crystallization temperature of 100°C for example, the enthalpies and the peak positions are independent of the heating rate (electronic supplementary material, figure S4 and table S1). Thus, the first hypothesis is excluded. For the second hypothesis, there was no other crystal form except the orthorhombic form from the WAXD results (as shown in figures 3 and 4). Therefore, the sequence of the double melting peak phenomenon could not have been caused by the different crystal forms.

**Table 2.** Melting data of HDPE/fibre composites.

crystallization temperature (°C)	*T*_m1_ (°C)	ΔH_1_ (J g^−1^)	*L*_1_ (nm)	*T*_m2_ (°C)	ΔH_2_ (J g^−1^)	*L*_2_ (nm)
110	131.2	2.5	18.7	127.1	2.3	14.4
100	130.1	4.8	17.3	126.4	9.4	13.9
90	129.4	5.0	16.5	125.6	9.8	13.3

**Figure 7.**
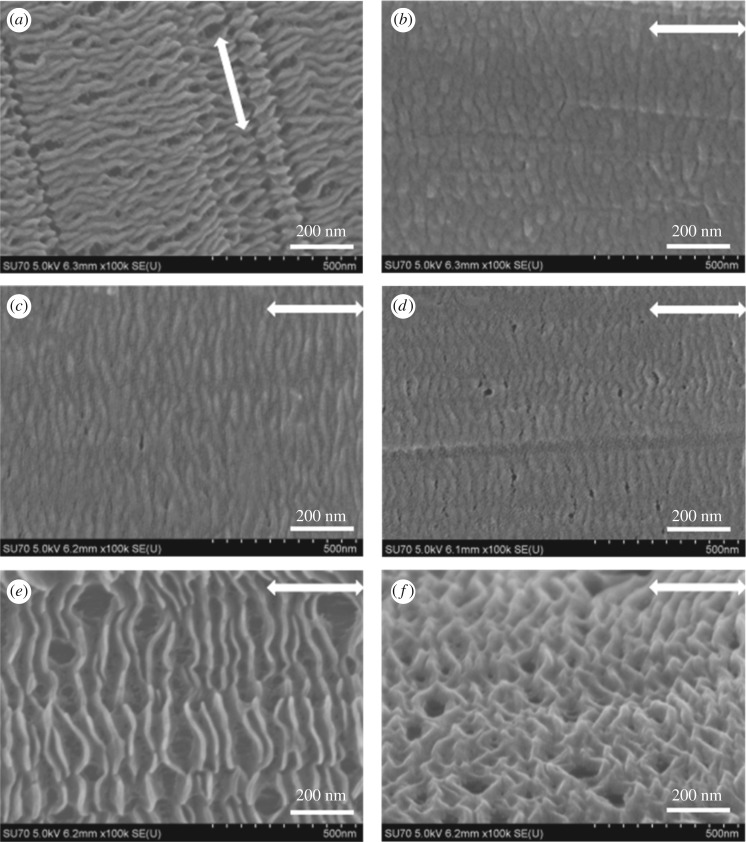
SEM micrographs of HDPE/UHMWPE fibre composites after being treated at 128°C for 3 min and then quenched to room temperature. Samples prepared at 110°C for 1 h are shown in (*a*) (before being treated) and (*b*) (after being treated). Samples prepared at 100°C and 90°C for 1 h are shown in (*c*) and (*d*), respectively. Samples prepared at 110°C for 3 h are shown in (*e*) (before being treated) and (*f*) (after being treated). The arrow represents the orientation of the UHMWPE fibre.

**Figure 8.**
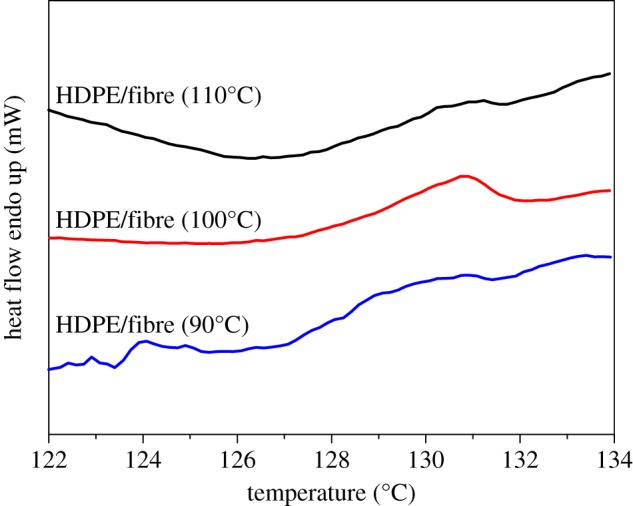
DSC first heating curves of HDPE/UHMWPE fibre composites treated at 128°C for 3 min and then quenched into iced alcohol.

To provide evidence for the third hypothesis of two kinds of crystals with different thicknesses, partial melting was applied to composites in order to further elucidate the fine structure of HDPE crystals induced by UHMWPE fibres. The initial composite samples (as in figures 1 and 2) were treated thermally at 128°C (which is close to the end of the lower melting peak, as shown in table 2) for 3 min and quenched into iced alcohol, producing samples for further analysis. It can be seen from the results shown in figure 7 that the ribbon crystals produced at 110°C for 1 h (figure 7*a*) transformed to brick-shaped crystals after being treated at 128°C for 3 min (figure 7*b*). This transformation was also observed in the other two samples after thermal treatment as shown in figure 7*c,d*. The fractionation of lamellae upon thermally treating them at higher temperatures indicates that the thermal stability along a coherent lamella is not uniform. Within a single lamella, some parts are more thermally stable or perfect than the others. The DSC heating curves of the samples after thermal treatment are shown in figure 8. Only one melting peak above 128°C was found, and the location was consistent with the higher one in figure 6. The average thicknesses of the brick crystals listed in table 3 are similar to *L*_1_ based on the calculation of the DSC higher melting peak (table 2). Therefore, all above-mentioned data lead us to draw the conclusion that the brick crystal remaining after melting at 128°C is the one corresponding to the higher melting temperature part. 2D-WAXD patterns of composites after treatment display two strong diffraction points, as shown in the electronic supplementary material, figure S5, and Herman's orientation parameter (*f*_c_) of all samples surpasses 97% (electronic supplementary material, figure S6), which is higher than that for samples before treated (less than 97% in figure 5*b*). This implies that the crystal structure of the lamellae near the fibre possesses a higher degree of ordering than the structure formed subsequently, which is more or less in line with the observations from SEM micrographs in figure 7 (after treated) and figure 1 (before treated), that is, the lamellar thickness decreases in an outward direction. Therefore, the inner lamellae are more thermally stable than those at the outer periphery. The composite sample produced at 90°C for 1 h has a low melting temperature and few crystals are left after being treated thermally at 128°C for 3 min (figure 7*d*), which subsequently results in low intensity of the melting peak in figure 8. According to the analysis of partial melting, the double melting peak phenomenon was caused by two crystals with different thicknesses: the crystals with high melting temperature grew close to the surface of the fibre, namely the ‘inner layer', which can also act as a heterogeneous nucleation site and afterwards induced the peripheral molecular chain to epitaxial crystallization on it. Consequently, the thinner and less-ordered crystals (outer layer) formed on the periphery.

**Table 3.** Average size of HDPE crystals formed on UHMWPE fibres after being treated thermally.

crystallization temperature (°C)	thickness of crystal (nm)	*d* (nm)^a^
110	20.1 ± 0.8	80 ± 7
100	18.2 ± 0.7	120 ± 11
90	17.4 ± 0.7	100 ± 9

^a^*d* is the lateral dimension of HDPE crystals formed on UHMWPE fibres after being treated thermally.

We can also see from figure 6 and table 2, for all composite samples, that the fusion enthalpies of the high melting peak (ΔH_1_, corresponding to the inner layer) are lower than those of the low melting peak (ΔH_2_, corresponding to the outer layer). This suggests that the lateral dimension of the brick crystals is small, which is also verified by the value obtained by SEM measurements in table 3, i.e. the lateral dimensions (*d*) of the inner layer are about 100 ± 9 nm, 120 ± 11 nm and 80 ± 7 nm, respectively, for 90°C, 100°C and 110°C, which are far below those of integral crystals formed on UHMWPE fibres for a crystallization period of 1 h (table 1). This also indicates that the formation of the inner lamellae blocked the strong induction from the highly oriented surface of the fibre, which provides a location for crystallization of a large number of molecules on the periphery. Owing to the crystal nucleus being less stable at lower degrees of undercooling, the lateral dimension of the crystals decreases when the isothermal crystallization temperature increases. Therefore, both ΔH_1_ and ΔH_2_ decrease as the isothermal temperature increases, which is in agreement with the SEM results shown in table 1 and table 3. In addition, the lateral dimension of the HDPE crystals directly induced by UHMWPE fibres (the inner layer) is approximately equal to that of polycaprolactone (PCL) epitaxial growth on the highly oriented PE substrate [38], which demonstrates that the vertical thickness of the ordering layer directly propagated from the interface of the highly oriented PE is about 100 nm. The difference is that the crystallization time of the HDPE molecular chains on UHMWPE fibres is less than an hour, which is far below that of PCL on highly oriented PE (half a month) due to the fast crystallization rate of HDPE. On the other hand, compared with the highly oriented PE substrate, the degree of orientation of UHMWPE fibres is much higher, and the ordered crystals induced can further act as an oriented substrate for epitaxial crystallization. The WAXD curves of samples after being treated thermally show that the two typical diffraction peaks at 2*θ* = 21.2° and 23.6° corresponding to the (110) and (200) crystal planes of the HDPE orthorhombic form remain unchanged. Thus, the second hypothesis mentioned above, i.e. there exist two different structures in the HDPE crystals formed on the surface of UHMWPE fibres, was further excluded here.

The composite samples crystallized for 3 h were also treated thermally at 128°C after 3 min, and the results are shown in figure 7*f*. Edge-on sheet shape crystals formed at 110°C after 3 h crystallization, as clearly shown in figure 7*e*, vanished and then transformed to a ribbon pattern after being treated at 128°C for 3 min (figure 7*f*). The ribbon pattern consists of the inner lamellae and amorphous layer produced by the melting of sheet shape crystals. Owing to the fact that the lateral dimension of sheet shape crystals after 3 h crystallization (figure 7*e* and table 1) is much larger than that after 1 h crystallization (figure 7*a* and table 1), the amorphous layer resulting from the melting of sheet shape crystals after 3 h crystallization is also much thicker and largely coats the inner lamellae and causes thicker lamellae in figure 7*f* than in figure 7*b*.

**Figure 9.**
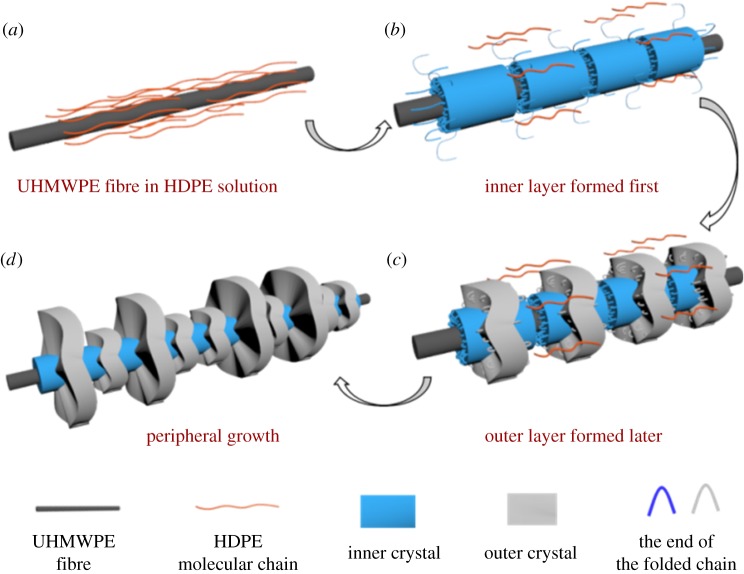
Schematic for the formation mechanism of the HDPE crystals induced by a UHMWPE fibre.

Based on the above findings, the development of the UHMWPE fibre-induced dual crystal structure is schematically depicted in figure 9. Owing to the smooth surface and the high degree of chain orientation and crystallization, the UHMWPE fibres has a high ability to induce polymer crystallization. And perfect lattice matching exists between UHMWPE fibres and HDPE because they contain the same polymer. Therefore, the surface of the UHMWPE fibre-induced HDPE molecular chain aligns along the fibre axis and forms brick-shaped thick crystals (inner layer in figure 9*b*). For a high density of active nuclei on the fibre surface, brick-shaped crystals packed closely and wrapped around the fibre. Away from the high orientation surface of the UHMWPE fibres, the inner lamellae act as heterogeneous nuclei to induce the an outer molecular chain to align and overgrow on the surface of them, forming an outer layer (figure 9*c*). Compared with the UHMWPE fibres with a high degree of chain orientation, the inner layer composed of folded chain crystals has a relatively lower regularity, which greatly affects the epitaxial crystallization of the outer molecular chain. Consequently, the thinner and distorted crystals constituted the outer layer (figure 9*c*), the distortion of which became much more significant with the increase in crystallization time, resulting in the formation of a curved sheet (figure 9*d*).

## 4. Conclusion

In this study, the components of HDPE crystals grown epitaxially on the surface of UHMWPE fibres were investigated by preparing their composites in solution. Owing to perfect lattice matching between UHMWPE fibres and HDPE, ribbon pattern crystals periodically decorated the UHMWPE fibres with the polymer chain parallel to the fibre axis in the early stages of crystallization, which then changes to a curved sheet shape as the crystallization time increases. The most suitable crystallization temperature for HDPE crystals moderately grown on fibres was 100°C; this is the temperature at which the largest size and most uniform lamellae formed. The orthorhombic crystal structure of HDPE remained unchanged in HDPE/UHMWPE fibre composite systems. Double melting peaks of the ribbon pattern crystal, which were ascribed to the bilayer components, were disclosed by partial melting experiments. That is, thicker and more regular brick-shaped crystals induced by a high degree of chain orientation on the UHMWPE fibres formed the inner layer, which subsequently induced the outer molecular chain to form a thinner and lower ordered crystal layer. A thorough analysis of epitaxial HDPE crystals on UHMWPE fibres in this research is expected to be helpful for understanding the evolution of crystal components from the interface to the periphery induced by a highly oriented substrate.

## Supplementary Material

Supporting information

## Data accessibility

The datasets supporting this article have been uploaded as part of the electronic supplementary material.
