# Supplementary material for: High-density polyethylene crystals with double melting peaks induced by ultra-high-molecular-weight polyethylene fibre
Source: R Soc Open Sci. 2018 Jul 18;5(7):180394. doi: 10.1098/rsos.180394 (PMC6083719; doi:10.1098/rsos.180394)
Supplement: Supporting information [file rsos180394supp1.docx]

Supporting information

**High density polyethylene crystals with double melting peaks induced by ultra-high molecular weight polyethylene fiber**

Weijun Miao^a^, Hao Zhu^a^, Tianchen Duan^a^, Hongbing Chen^a^, Feng Wu^a^, Libin Jiang^a^, Zongbao Wang^a,*^

Figure S1 shows the SEM micrograph of UHMWPE fiber. It can be observed that the virgin UHMWPE fiber has a neat surface, and the diameter of UHMWPE fiber ($D_{fiber}$) measured is 17.2 µm.


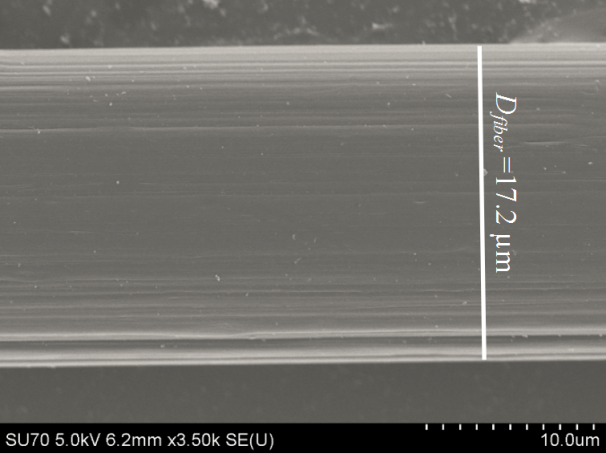


**Figure S1**. SEM micrograph of UHMWPE fiber. The white line represents the diameter of UHMWPE fiber.

Figure S2 shows that the 2D WAXD pattern of HDPE/UHMWPE fiber composite produced at 100 ^o^C for 3 h. The size of arcs is larger evidently than that for 1h (Figure 3d), and the Herman’s orientation parameter (*f_c_*) is 84.5%, significantly lower than 95.3% for 1h (Figure 5b). This suggests that the orientation of the crystal becomes worse with increasing the crystallization time.


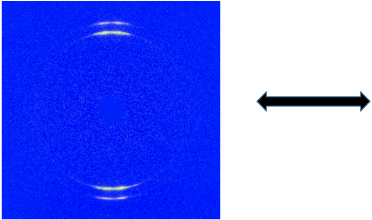


**Figure S2**. 2D WAXD pattern of HDPE/UHMWPE fiber composite produced at 100 ^o^C in p-xylene for 3 h. The arrow represents the orientation of the UHMWPE fiber.

As shown in Figure S3, The DSC heating curve of UHMWPE fiber presents two obvious melting peaks.The first melting peak between 145 ^o^C is caused by the melting of micro-fibrils crystals, and the second melting peak located between 152 ^o^C is the result of the orthorhombic to hexagonal phase transition. High degree of chain orientation in UHMWPE fiber contributes to the higher melting point compared with bulk HDPE (131 ^o^C).

**Figure S3.** DSC heating curves of neat HDPE and UHMWPE fiber.

Figure S4 shows the DSC first heating curves recorded at increasing heating rates for HDPE/UHMWPE fiber composite produced at 100 ^o^C in p-xylene for 1 h. Melting data are listed in Table S1. Obviously, the enthalpies and the peak positions basically remain unchanged as increasing the heating rate.

**Figure S4.** DSC first heating curves recorded at increasing heating rates for HDPE/UHMWPE fiber composite produced at 100 ^o^C in p-xylene for 1 h.

**Table S1.** Melting data of HDPE/Fiber composites measured at different heating rates

| heating rate (^o^C/min) | T_m1_(^o^C) | ΔH_1_(J/g) | T_m2_(^o^C) | ΔH_2_(J/g) |
| --- | --- | --- | --- | --- |
| 2 | 129.9 | 4.9 | 126.2 | 9.4 |
| 10 | 130.1 | 4.8 | 126.4 | 9.4 |
| 40 | 130.1 | 4.8 | 126.5 | 9.3 |

As shown in Figure S5, 2D-WAXD patterns of composites after treated display two strong diffraction points comparable with neat UHMWPE fiber, which indicate extremely high chain orientation along fiber axis.


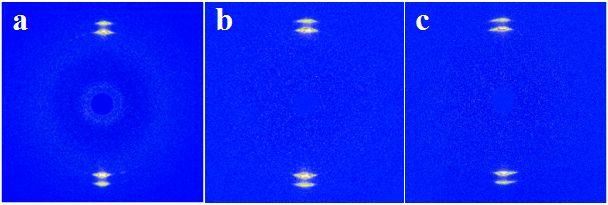


**Figure S5.** 2D WAXD patterns of HDPE/UHMWPE fiber composites produced at (a) 110 ^o^C, (b) 100 ^o^C, and (c) 90 ^o^C in p-xylene for 1 h and followed by treating at 128 ^o^C for 3 min.

Figure S6 shows that the Herman’s orientation parameter (*f_c_*) of fiber composites after thermal treatment. The Herman’s orientation parameters of all samples surpass 97%, higher than that of samples before treated (<97% in Figure 5b), although they decrease when isothermal crystallization temperature increases. This suggests the exceptionally high orientation of inner crystal.

**Figure S6.** The degree of crystal orientation in fiber composites after treated at 128 ^o^C for 3 min.
